# Kinesiophobia, Physical Limitations and Psychological Distress as Barriers to Physical Activity in Heart Transplantation Patients: A Qualitative Study

**DOI:** 10.3390/jcm14217867

**Published:** 2025-11-05

**Authors:** Elena Marques-Sule, Juan Luis Cabanillas-García, Luis Almenar-Bonet, Amalia Sillero-Sillero, Maria Cruz Sánchez-Gómez, Raquel Ayuso-Margañon, Raquel López Vilella, Noemí Moreno-Segura

**Affiliations:** 1 Physiotherapy in Motion, Multispeciality Research Group (PTinMOTION), Department of Physiotherapy, University of Valencia, 46010 Valencia, Spain; elena.marques@uv.es; 2 Department of Physiotherapy, Faculty of Physiotherapy, University of Valencia, 46010 Valencia, Spain; noemi.moreno@uv.es; 3 Department of Educational Sciences, Area of Methods and Diagnosis in Education, University of Extremadura, 06011 Badajoz, Spain; juanluiscg@unex.es; 4 Heart Failure and Transplantation Unit, Department of Cardiology, Hospital Universitario y Politécnico La Fe, 46026 Valencia, Spain; lualmenar@gmail.com (L.A.-B.); raqvil@gva.es (R.L.V.); 5 Biomedical Research Network Center for Cardiovascular Diseases (CIBERCV), Carlos III Health Institute, 28029 Madrid, Spain; 6 Department of Medicine, University of Valencia, 46010 Valencia, Spain; 7 Escola Universitària Gimbernat, Adscrita a Universitat Autònoma de Barcelona, 08174 Sant Cugat, Spain; 8 Department of Didactics, Organization and Research Methods, University of Salamanca, 37008 Salamanca, Spain; mcsago@usal.es; 9 Hospital del Mar Nursing School (ESIHMar), Universitat Pompeu Fabra-Affiliated, C/Doctor Aiguader, 80, 08003 Barcelona, Spain; rayuso@esimar.edu.es; 10 Social Determinants and Health Education Research Group (SDHEd), Hospital del Mar Research Institute, 08003 Barcelona, Spain

**Keywords:** heart transplantation, kinesiophobia, physical activity, rehabilitation, patient experience, qualitative research

## Abstract

**Background/Objectives**: Heart transplantation substantially improves survival and quality of life in patients with advanced heart failure; however, many heart transplantation patients fail to recover normal physical activity levels. Persistent inactivity compromises secondary prevention and long-term outcomes. Kinesiophobia—an excessive and irrational fear of movement—may act as a central barrier limiting physical activity after heart transplantation. This study aimed to explore how kinesiophobia develops and interacts with physical and psychological factors that influence adherence to an active lifestyle after heart transplantation. **Methods:** A qualitative study was conducted in 24 adult heart transplantation patients (mean age 62.1 years; 83% male) at a tertiary hospital in Spain. Semi-structured interviews lasting 35–60 min were transcribed verbatim and analysed using reflexive thematic analysis. Methodological rigour was ensured through triangulation, reflexivity, and transparent documentation of analytic decisions. **Results**: Three interrelated themes were identified: (1) Kinesiophobia, characterised by fear of overexertion and avoidance of performing physical activity; (2) physical limitations, including fatigue, muscle weakness, treatment side effects, and intensified perceptions of vulnerability; and (3) psychological distress, encompassing anxiety, demotivation, and frustration, which intensified inactivity. These domains formed a self-perpetuating cycle that restricted participation in physical activity. Some participants reported simple adaptive strategies, such as pacing, walking and social support that enhanced their sense of safety and confidence. **Conclusions**: Kinesiophobia, physical limitations, and psychological distress interact to restrict physical activity in heart transplantation patients. Our findings suggest that rehabilitation should integrate psychological support, cognitive-behavioural strategies, and tailored education to reduce fear, enhance self-efficacy, and promote sustainable physical activity engagement.

## 1. Introduction

Heart transplantation (HTx) remains the treatment of choice for patients with advanced heart failure, providing substantial improvements in survival, quality of life, and exercise tolerance [1,2]. Despite these benefits, the exercise capacity of recipients remains markedly impaired, reaching only around 50% of that observed in age- and sex-matched healthy individuals one year after surgery [2]. This limitation has significant clinical implications, as insufficient physical activity (PA) is strongly associated with poorer long-term outcomes, higher morbidity, and reduced quality of life [3]. Understanding the multidimensional reasons for persistent inactivity, including psychological, physical, and emotional factors, is therefore crucial to secondary prevention in HTx patients.

Several mechanisms may contribute to this limitation. Multiple physiological, psychological, and emotional factors contribute to this limitation [4,5,6]. Understanding their interaction is essential for effective rehabilitation and to promote PA. Among these, kinesiophobia—defined as an excessive or irrational fear of movement that leads to avoidance of activity [5,6,7]—is increasingly recognised as a relevant psychological barrier after HTx [8]. In these patients, it interacts with physiological factors (such as cardiac denervation and immunosuppressive side effects) and psychological burden, amplifying avoidance behaviours.

Although barriers to physical activity after HTx have been investigated, the specific role of kinesiophobia and its interaction with other physical and emotional factors remains insufficiently understood. In the broader context of cardiac rehabilitation, addressing kinesiophobia is essential to promote adherence to exercise and long-term recovery [9,10]. Cardiac rehabilitation programmes that include psychological support and patient education have been shown to reduce fear of movement and improve participation in other cardiac populations. However, evidence in this regard remains scarce in HTx patients.

Evidence highlights the need for a biopsychosocial approach to address inactivity, recognising psychological factors such as kinesiophobia as significant barriers to adherence [8,9,10]. Although qualitative work has explored general PA facilitators and barriers after HTx [11], the explicit role of kinesiophobia, and its interplay with physical limitations and emotional distress, remains insufficiently understood in rehabilitation planning. To address this gap, a deeper understanding of the lived experiences of patients is required.

Recent quantitative findings have demonstrated that high levels of kinesiophobia in HTx patients are associated with greater disability, lower self-efficacy, and reduced extrinsic motivation to perform PA [12]. While these results confirm its clinical relevance, quantitative designs cannot capture the multidimensional lived experience, particularly the emotional and cognitive processes through which patients interpret vulnerability. A qualitative approach is therefore needed to explore how these dimensions converge in everyday life after HTx.

Qualitative research offers a robust framework to capture these perspectives, yielding insights into how kinesiophobia is experienced in daily life, how it influences adherence to PA, and how it shapes recovery trajectories [13]. Such understanding is critical to inform multidisciplinary rehabilitation programmes that address psychological, physical, and emotional barriers concurrently.

Accordingly, this study aimed to explore how kinesiophobia develops and operates as a central barrier to performing PA in HTx patients, and how it interacts with physical, psychological and emotional factors influencing adherence to an active lifestyle.

## 2. Materials and Methods

### 2.1. Study Design and Analytical Framework

We conducted a qualitative study grounded in an interpretivist paradigm. This design was chosen to capture the lived experiences of HTx regarding PA and kinesiophobia, acknowledging the subjective meanings patients assign to recovery and adaptation. A qualitative interpretive approach was considered the most suitable for addressing the psychological and physical dimensions of post-transplant life, which are not easily quantifiable. Data analysis followed Reflexive Thematic Analysis (RTA), as described by Braun and Clarke [14,15], a methodology that enables the identification of patterns of meaning across narratives while maintaining sensitivity to the context of preventive cardiology and behavioural rehabilitation. RTA was selected because it provides an analytical framework well-suited to examining the interplay of psychological barriers, physical limitations, behavioural adaptations and psychological burden after transplantation. Consistent with a reflexive stance, researchers engaged in iterative meaning-making rather than treating coding as a reliability exercise.

### 2.2. Participants and Setting

The study population was drawn from the established cohort of HTx patients attending structured, multidisciplinary post-transplant care at the Hospital Universitario y Politécnico La Fe (Valencia, Spain), a tertiary referral centre with a consolidated HTx programme. Recruitment was conducted between November 2022 and February 2023 using a purposive sampling strategy designed to capture maximum variation in key demographic and clinical characteristics (e.g., age, sex, and time since transplantation). Accessibility of the existing clinical cohort facilitated recruitment, but selection was primarily based on conceptual relevance rather than convenience. This strategy sought conceptual breadth to achieve interpretive depth regarding kinesiophobia and co-occurring barriers.

Eligible participants were adults aged 18 years or older, with stable graft function confirmed during outpatient cardiology consultations, able to communicate in Spanish, and willing to provide written informed consent. Exclusion criteria included severe psychiatric illness interfering with comprehension, cognitive impairment precluding participation, or refusal to provide consent. Clinical variables (e.g., immunosuppressive regimen, comorbidities) were noted to contextualise perceived and actual PA level.

Recruitment proceeded until theoretical data saturation was reached, defined as the stage at which no new conceptual categories or variations emerged despite additional interviews [16]. Saturation was discussed iteratively in team meetings; the final sample (n = 24) was judged adequate for thematic sufficiency and variance within an HTx cohort focused on PA barriers.

### 2.3. Data Collection

Data were collected through semi-structured, in-depth interviews conducted in private consultation rooms to ensure confidentiality and minimise external influences. Each interview lasted between 35 and 60 min (mean: 47 min). An interview guide, developed based on prior literature and clinical expertise, explored patients’ experiences of PA, perceptions of safety and risk, psychological responses to PA, and perceived facilitators and barriers. Open-ended questions encouraged detailed narratives, while targeted probes addressed fear, physical limitations, and adaptation strategies. All interviews were audio-recorded with consent, transcribed verbatim, anonymised, and verified for accuracy. To enhance replicability, the interview guide, data collection conditions, and procedures were applied consistently across participants. Field notes were taken immediately after each interview to capture contextual information and the researcher’s reflections. The interview guide is shown in Table 1.

### 2.4. Data Analysis

Interview transcripts were analysed following Braun and Clarke’s six-phase framework for RTA [14,15]. The process included immersion in the data, generation of initial codes, organisation into candidate themes, review and refinement of themes, definition and naming of final themes, and production of an analytic narrative that integrated participants’ quotations with interpretative synthesis. Two researchers independently coded the data using NVivo 14 software (QSR International Pty Ltd., Burlington, MA, USA) [17], followed by interpretive consensus meetings that prioritised reflexive discussion over agreement metrics. Additional researchers acted as critical reviewers. In accordance with RTA principles, coding was not treated as a reliability exercise, and inter-rater agreement statistics were not applied. Instead, analytic rigour was ensured through reflexive dialogue, transparent documentation of coding decisions, and iterative team discussions. Themes were structured hierarchically, comprising three overarching themes and seven subthemes, which captured the interaction between kinesiophobia and physical/physiological and emotional constraints that shape PA engagement after HTx. Representative quotations were selected to illustrate findings and preserve the authenticity of participants’ voices. Abridged theme maps and an audit of coding decisions were retained in the analytic trail.

### 2.5. Rigour and Reflexivity

Methodological rigour was ensured in accordance with Lincoln and Guba’s criteria of trustworthiness [18]. Credibility was supported by investigator triangulation, prolonged engagement with the data, inclusion of representative quotations, and theoretical saturation, defined as the stage at which no new categories emerged. Transferability was addressed by providing detailed contextual descriptions of the clinical setting, recruitment procedures, and participant characteristics, allowing readers to assess the applicability of the findings to other contexts. Dependability was ensured by maintaining a transparent audit trail documenting coding decisions, theme evolution, and analytic reflections. Confirmability was reinforced through reflexive journaling, peer debriefing, and systematic grounding of interpretations in the dataset. Reflexivity was integrated throughout, as positionality notes examined how cardiology, physiotherapy, and nursing backgrounds, as well as expectations about “safe” exertion, medication effects, and fear-avoidance, might shape interpretation. These reflections were discussed in scheduled debriefs and logged in the audit trail. Reflexive memos and structured team debriefings enhanced transparency, acknowledged the subjectivity inherent to qualitative research, and strengthened the robustness of analysis. In addition, researchers explicitly considered how their professional backgrounds (cardiology, physiotherapy, nursing) might shape interpretation and engaged in ongoing positionality reflection to minimise undue influence.

### 2.6. Ethical Considerations

The study was conducted in accordance with the Declaration of Helsinki [19] and approved by the Institutional Review Board of the Hospital Universitario y Politécnico La Fe (approval code: (IE2090936)/2 June 2022). All participants provided written informed consent before data collection. Confidentiality and data security were preserved through anonymisation of transcripts, restricted access to files, and compliance with data protection regulations.

## 3. Results

### 3.1. Sociodemographic Characteristics

The sample included 24 HTx patients (mean age: 62.1 ± 14.0 years; 83.3% male). Most participants were married (71%), whilst 92% were retired, and 63% had completed primary education. Average time since transplantation was 13.2 ± 8.3 years. Table 2 summarises the demographic and clinical characteristics of the sample.

### 3.2. Thematic Findings

Thematic analysis identified three main themes and seven subthemes that explain how HTx patients experience PA. These findings highlight the multidimensional interplay of Kinesiophobia, physical limitations, and psychological distress in shaping recovery trajectories, underscoring the need for targeted clinical interventions. Table 3 summarises the themes and subthemes identified in the analysis.

#### 3.2.1. Kinesiophobia

Kinesiophobia emerged as the predominant barrier, with patients interpreting physical exertion as a potential threat to the transplanted heart. This anticipatory fear fostered avoidance, reducing engagement in PA and undermining the protective cardiovascular effects of PA.

##### Perception of Movement as a Threat

Participants described uncertainty about their physical limitations, often shaped by previous negative experiences. Fear of harming the graft or provoking symptoms created a cycle of avoidance of performing PA.

*“What worries me is that my heart might stop beating if I push myself too hard when doing PA.” (P4)*

*“I don’t know what my body can handle anymore. That uncertainty makes me hesitant even to try.” (P13)*

*“I had a bad experience. I felt faint after a workout, and since then, I am scared to push myself.” (P8)*

##### Feeling Safe Enough to Perform PA

Despite their fears, many patients adopted adaptive strategies, such as switching to low-impact PA, performing PA at home, or seeking support from relatives. These adjustments illustrate attempts to manage kinesiophobia by regaining a sense of safety.

*“I feel safer doing PA at home because I can stop whenever I need to.” (P3)*

*“I’ve stopped doing high-impact activities like running, but I can walk, and that makes me feel more comfortable.” (P13)*

*“My wife goes for a walk with me every day. Knowing she’s there makes me feel better.” (P23)*

#### 3.2.2. Impact of Transplantation on PA

Although HTx restored survival and stability, physical limitations and medication side effects continued to constrain activity. These barriers interacted with psychological fears, amplifying perceptions of fragility.

##### Physical Limitations

Persistent fatigue, weakness, and musculoskeletal pain limited performance, forcing patients to adjust expectations and reduce intensity. This mismatch between expected recovery and lived experience reinforced caution.

*“After the surgery, I was not able to do anything until three months later.” (P4)*

*“Before, I even played tennis, but after the transplant, I am not able to continue due to the required high intensity.” (P22)*

*“Climbing stairs became a challenge. I never thought it would be so hard after the transplant.” (P11)*

##### Drug Side Effects

Corticosteroid-related swelling, fluid retention, and weight gain were widely reported, contributing to discomfort and discouragement. Medication not only reduced capacity but also reinforced the perception of vulnerability.

*“When I underwent the surgery, I felt bloated due to cortisone, which hindered my ability to perform PA.” (P21)*

*“Medication makes me feel heavy, and sometimes it’s just too uncomfortable to move.” (P8)*

#### 3.2.3. Psychological Impact of the Transplant

The transplant experience also generated psychological consequences that discouraged performing PA, including reduced motivation, anxiety, and emotional distress. These factors compounded physical barriers, resulting in conservative attitudes towards exertion.

##### Reduced Motivation

Patients described a progressive loss of motivation, often related to fatigue and perceived lack of progress. This emotional exhaustion reinforced avoidance behaviours.

*“I am thinking about this problem a lot; every day I am less motivated, and I have less strength.” (P15)*

*“Emotionally, I feel worse and less motivated. I think about my status when I can’t perform PA like I did before.” (P21)*

##### Anxiety and Stress

Anticipatory anxiety about complications or overexertion frequently intensified fear, sometimes manifested as somatic symptoms.

*“I had an anxiety crisis because I was nervous about not being able to do PA.” (P16)*

*“The other day, my heart hurt, but the doctors said it was just anxiety and stress.” (P4)*

##### Other Emotional Factors

Feelings of frustration, sadness, and dependence emerged as recurrent concerns, often linked to family responsibilities and uncertainty about the future (Table 4).

*“I felt frustrated the first month when I was discharged from the hospital, and I felt so dependent.” (P14)*

*“I am very sad due to the transplant because I have six children, and I am worried about their future.” (P1)*

## 4. Discussion

This qualitative study offers novel insights into the multidimensional barriers that may affect performing PA in HTx patients. Reflexive thematic analysis identified three interrelated themes, kinesiophobia, physical limitations, and psychological and emotional distress, that together restrict patients’ ability to benefit from performing PA as a preventive cardiovascular strategy after HTx.

### 4.1. Kinesiophobia and the Fear-Avoidance Cycle

Kinesiophobia emerged as the predominant psychological barrier to performing PA. Participants frequently perceived movement as a threat, expressing uncertainty about their physical limitations and fearing potential harm to the transplanted heart. These fears triggered avoidance behaviours, reinforcing a cycle of inactivity, deconditioning, and emotional distress. This pattern aligns with the fear-avoidance model [20,21] and with evidence showing that perceived danger perpetuates physical inactivity and adverse outcomes in cardiac and transplant populations [22].

Our findings expand on previous qualitative research in HTx patients [23,24,25], illustrating how fear is not only linked to physical harm but also to loss of autonomy, disruption of self-identity, and anxiety about the future. Unlike Stubber et al. [25], who emphasised uncertainty around medical management, our findings highlight kinesiophobia as a central theme shaping daily activity choices. Similarly, while Fatma et al. [24] described the challenges of adjusting to “a new life”, our study adds the anticipatory dimension of fear, linking it directly to the avoidance of PA, underscoring its psychological and social impact, and requiring targeted interventions.

### 4.2. Physiological Constraints and Interaction with Psychological Fear

Physical barriers, such as fatigue, muscle weakness, and joint pain, were commonly reported. These findings align with existing evidence documenting reduced PA after HTx [26]. Corticosteroid-related side effects, including swelling, weight gain, and fluid retention, further limited PA and reinforced perceptions of fragility [27,28,29].

In addition, cardiac denervation after HTx alters autonomic regulation during exertion, removing familiar cues such as heart rate acceleration [26,30]. This uncertainty intensifies kinesiophobia, as patients struggle to gauge the level of PA in a safe range. Unlike prior quantitative work [24], our study highlights the lived experience of this physiological–psychological interplay, offering a richer context for clinical decision-making.

### 4.3. Coping Strategies and Preventive Opportunities

Despite these barriers, many participants adopted adaptive strategies such as walking, stretching, and pacing their daily routines—behaviours consistent with graded PA approaches used in interventions targeting cardiac patients [31]. Social support emerged as a key facilitator, enhancing safety perceptions and PA performance, consistent with evidence showing that peer and family involvement improve confidence and long-term participation [32,33,34].

Our findings suggest that HTx patients should combine supervised PA with psychosocial interventions to reduce fear and promote the performance of PA. Education addressing misconceptions, cognitive-behavioural therapy, and motivational interviewing have proven effective in cardiac populations [35], and may be promising tools for systematic incorporation in interventions targeted to HTx patients.

### 4.4. Emotional Burden and Gender Differences

Emotional distress, reduced motivation, anxiety, sadness, and worry about the future were consistent findings, echoing previous reviews on the psychosocial burden of transplantation [36,37]. While gender-related differences were not a primary focus of this study, exploratory observations suggest that female participants may experience a higher psychological burden and greater kinesiophobia. However, given the small sample size of women in our research, these findings should be interpreted with caution and cannot be generalised. Further research with larger and more gender-balanced samples is needed to confirm these patterns and inform gender-sensitive rehabilitation strategies.

### 4.5. Strengths and Limitations

This study offers an in-depth, clinically oriented exploration of HTx patients’ experiences with PA, providing interpretive insights beyond descriptive accounts. The rigorous application of reflexive thematic analysis [15], triangulation, and adherence to trustworthiness criteria [18] enhances the robustness of the findings. Importantly, this is one of the few qualitative studies to focus specifically on kinesiophobia in HTx patients, representing a novel contribution to previous literature. The integration of reflexivity and comparison with existing qualitative studies further reinforces methodological transparency and interpretive depth.

However, limitations must be acknowledged. The study was conducted in a single centre with a relatively small, predominantly male sample, which may limit its transferability. As a cross-sectional qualitative design, it captures perceptions at one point in time and may not reflect longitudinal changes in attitudes towards PA. Including long-term survivors (≥20 years post-transplantation in some cases) provides valuable insight into chronic adaptation but may not reflect the experiences of more recent recipients. Nonetheless, achieving theoretical saturation [16] strengthens the credibility of the findings and supports the relevance of the identified themes for guiding clinical practice. Additionally, recruiting from a specialised tertiary centre may introduce selection bias, as participants could reflect a particularly adherent and clinically stable subgroup. Multicentre studies with more diverse cohorts are needed to assess transferability.

### 4.6. Clinical Implications

The findings have direct implications for clinical practice. Interventions targeted to HTx patients should be approached as multidimensional processes which should address psychological, physical, and social barriers simultaneously.

Psychological interventions, including cognitive-behavioural therapy, motivational interviewing, and graded exposure, may help to reduce kinesiophobia and enhance self-efficacy [35]. Although supported in broader cardiac populations, the application of these psychological interventions in HTx should be further investigated in mixed-methods or experimental research.

Multidisciplinary interventions that combine psychological support with supervised PA are promising in other cardiac populations and could be adapted for HTx patients to improve several outcomes, as described in the present study. Low-impact, supervised PA interventions should be introduced progressively, with monitoring by multidisciplinary teams [31]. Social support through family involvement and peer support may enhance confidence, reduce fear, and improve PA performance [32,33,34], although further evidence is needed in this regard.

Gender-sensitive care should address psychosocial differences to optimise recovery. Given the limited representation of women in this study, these suggestions must be interpreted with caution, and further research should focus on developing evidence-based, gender-sensitive interventions.

Together, these measures may help transform post-transplant interventions into preventive strategies that promote PA performance in HTx patients while enhancing psychological resilience and long-term quality of life.

## 5. Conclusions

Kinesiophobia, physical limitations, and psychological distress interact to restrict PA in HTx patients, creating a cycle of inactivity and emotional burden. Addressing misconceptions, integrating psychological support, and leveraging social support may improve the performance of PA in HTx patients. Nevertheless, further research is needed to confirm these strategies.

This study highlights the potential value of multidisciplinary, patient-centred interventions that simultaneously address physical and psychological barriers. Such approaches may help healthcare teams to support cardiovascular protection and enhance the long-term quality of life of HTx patients. However, these findings should be interpreted with caution and considered as preliminary insights.

## Figures and Tables

**Table 1:** Semi-structured interview guide.

No.	Core Question
1	What do you like or value about physical activity (PA)?
2	How do you usually perform PA? Why do you engage (or not) in these activities?
3	What attitude do you have toward PA after the transplant?
4	Have you ever avoided PA because of pain or fear of worsening your condition?
5	What situations or feelings make you more afraid of moving or exercising?
6	What strategies help you feel safe or confident when being active?
7	How do you feel when you perform PA (emotionally, physiologically and physically)?
8	What could help you maintain regular PA in the future?

**Table 2:** Demographic and clinical characteristics of the sample.

Variable	Mean ± SD/Frequency (Percentage)
**Demographic Characteristics**	
**Sex**	
Male	20 (83.33%)
Female	4 (16.67%)
**Age (years)**	62.08 ± 14.02
**Marital Status**	
Married	17 (70.8%)
Divorced	3 (12.5%)
Single	3 (12.5%)
Widow	1 (4.17%)
**Working Status**	
Retired	22 (91.67%)
Worker	1 (4.17%)
Student	1 (4.17%)
**Education Level**	
Primary Education	15 (62.5%)
Secondary Education	6 (25%)
University Education	3 (12.5%)
**Clinical Characteristics**	
**Time Since Transplantation (years)**	13.27 ± 8.26

Data showed in mean ± SD or in frequency (%) as appropriate.

**Table 3:** Themes and subthemes identified through reflexive thematic analysis.

Key Themes	Subthemes
1. Kinesiophobia	1.1. Perception of Movement as a Threat 1.2. Feeling Safe Enough to Perform PA
2. Impact of Transplant on PA	2.1. Physical Limitations 2.2. Drug Side Effects
3. Psychological Impact of the Transplant	3.1. Reduced Motivation 3.2. Anxiety and Stress 3.3. Other Emotional Factors

Abbreviation: PA = physical activity.

**Table 4:** Summary of barriers and facilitators to physical activity after heart transplantation.

Barriers to Perform PA	Facilitators to Perform PA
Kinesiophobia and fear of overexertion	Psychological support (motivational interviewing)
Fatigue, muscle weakness, joint pain	Adaptive strategies (e.g., walking, stretching)
Corticosteroid side effects (swelling, weight gain)	Graded activity and pacing routines
Cardiac denervation and lack of heart rate cues	Supervised PA and tailored interventions
Emotional distress and perceived vulnerability	Social support (family, peers)
Misconceptions about safety when performing PA	Education and reassurance from healthcare professionals

Abbreviation: PA = physical activity.

## Data Availability

Aggregated data supporting this study’s findings are available upon reasonable request from the corresponding author, A.S.S., subject to review. These data are not publicly available due to privacy concerns and the potential for compromising research participant privacy/consent.

## Abbreviations

The following abbreviations are used in this manuscript:

HTx	Heart transplantation
PA	Physical activity
RTA	Reflexive thematic analysis
CBT	Cognitive-behavioural therapy
ISHLT	International Society for Heart and Lung Transplantation
PPE	Personal protective equipment
